# AKI in a Patient with Fatigue, Weakness, and an Active Urine Sediment

**DOI:** 10.34067/KID.0000000000000083

**Published:** 2023-04-27

**Authors:** Percy Adonteng-Boateng, Meghan E. Kapp

**Affiliations:** 1 Division of Nephrology, Department of Medicine, University Hospitals, Cleveland, Ohio; 2 Department of Pathology, University Hospitals, Case Western Reserve University, Cleveland, Ohio

**Keywords:** clinical nephrology, anti-GBM, crescents, giant cells, GN, immunofluorescence, light microscopy

## Abstract

**Figure:**
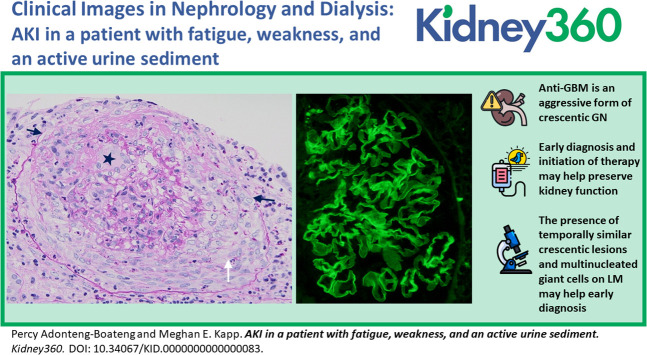

## Case Description

A 53-year-old woman presented with a 2-week history of progressive general weakness and fatigue associated with poor oral intake, nausea, and intermittent vomiting. She denied fever, rash, and hematuria. She reports no smoking, alcohol, recreational drug, or non-steroidal anti-inflammatory drug (NSAID) use. She had no family history of autoimmune disease. On physical examination, she appeared fatigued with bilateral lower extremity pitting edema (1+) and stable vital signs. Laboratory results showed mild leukocytosis, microcytic anemia, a normal platelet count, and elevated serum creatinine (4.9 mg/dl). Urinalysis was significant for hematuria, proteinuria (urine protein creatinine ratio of 1 g), and pyuria. Complements C3 and C4 were normal. While ANCA and antiglomerular basement membrane (GBM) titers were pending and with worsening kidney function, the patient underwent a kidney biopsy.

**Figure 1:**
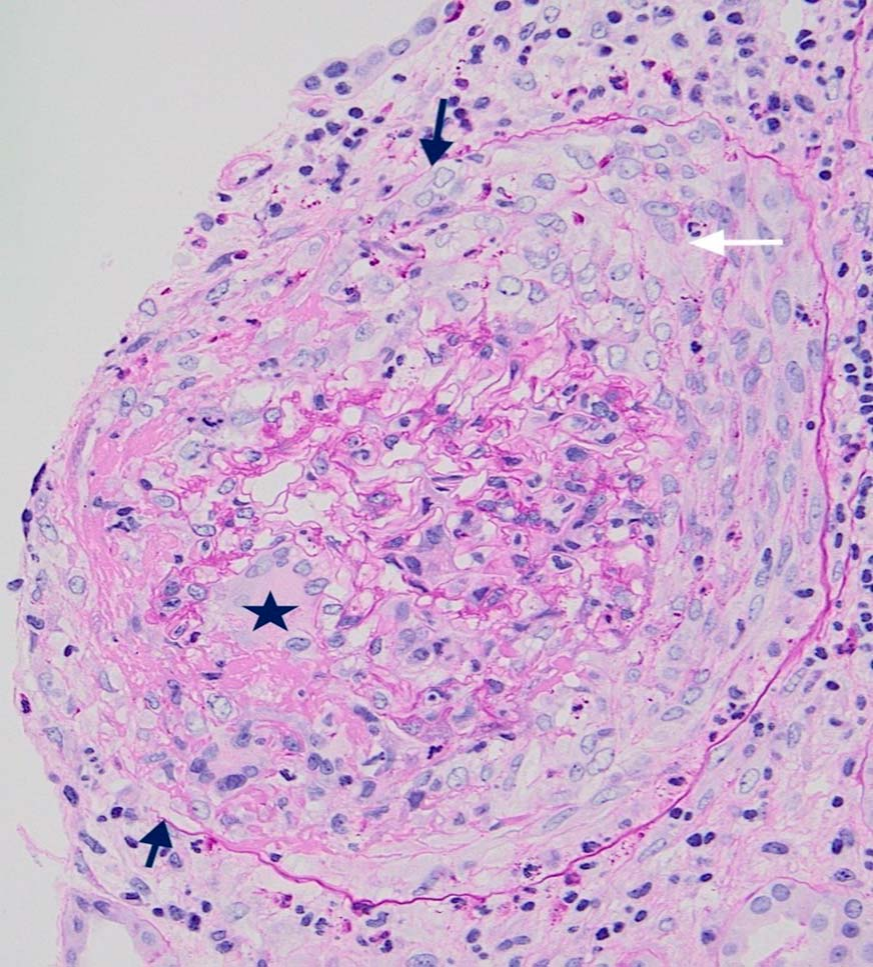
**A glomerulus with GBM break, cellular crescent in Bowman's space (white arrow), multinucleated giant cell (star), and Bowman’s capsule rupture (black arrow) (PAS; 400×).** PAS, periodic acid–Schiff.

**Figure 2:**
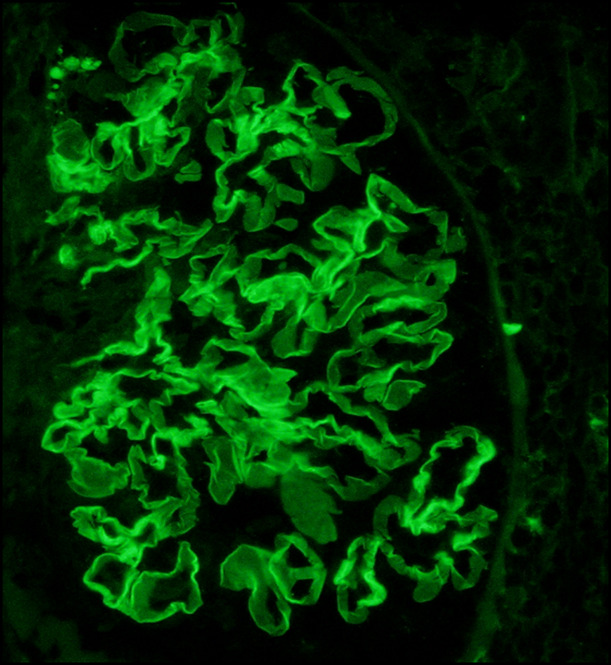
Immunofluorescence shows polyclonal IgG linear staining of GBMs (anti-IgG antibody; 400×).

Kidney biopsy revealed a diffuse necrotizing crescentic GN without endocapillary hypercellularity. Rare glomeruli contained multinucleated giant cells (Figure 1). Immunofluorescence demonstrated polyclonal immunoglobulin G (IgG) linear staining of GBMs (Figure 2) without deposits on electron microscopy. These findings are indicative of anti-GBM antibody-mediated GN. Her anti-GBM titer was subsequently found to be markedly elevated at 258 (normal being undetectable).

## Discussion

Anti-GBM antibody-mediated GN (anti-GBM) is a rare form of small vessel vasculitis caused by autoantibodies against the noncollagenous domain (NC 1) of the alpha 3 chain of type IV collagen. The disease presents with rapidly progressive crescentic GN.^1^ Early diagnosis and initiation of therapy may help preserve kidney function. Therapy includes aggressive removal of the autoantibodies with plasma exchange, halting antibody production with immunosuppression, and supportive care with renal replacement therapy.^2^ Delay in diagnosis and initiation of therapy may be encountered in situations where serologic tests are lacking, and tissue is not available for immunofluorescence to help distinguish anti-GBM from other causes of crescentic GN, such as immune-complex and ANCA-associated vasculitis. Recognizing light microscopic (LM) features that may favor anti-GBM may go a long way in making early diagnosis/initiation of therapy. Such findings include absence of endocapillary hypercellularity, same age of glomerular lesions, and presence of multinucleated giant cells. Multinucleated giant cells are reportedly more common in anti-GBM than ANCA-associated GN and underscore the explosive nature and rapid destruction of glomeruli seen in anti-GBM.^3,4^

## Teaching Points

- Anti-GBM is an aggressive form of crescentic GN.
- Early diagnosis and initiation of therapy may help preserve kidney function.
- The presence of temporally similar crescentic lesions and multinucleated giant cells on light microscopy may help early diagnosis.
